# Supplementary figures and images for: Tau oligomers modulate synapse fate by eliciting progressive bipartite synapse dysregulation and synapse loss
Source: Mol Neurodegener. 2026 Jan 22;21:13. doi: 10.1186/s13024-026-00928-2 (PMC12918473; doi:10.1186/s13024-026-00928-2)

Figure 1D

Blot #1: anti-Streptavidin

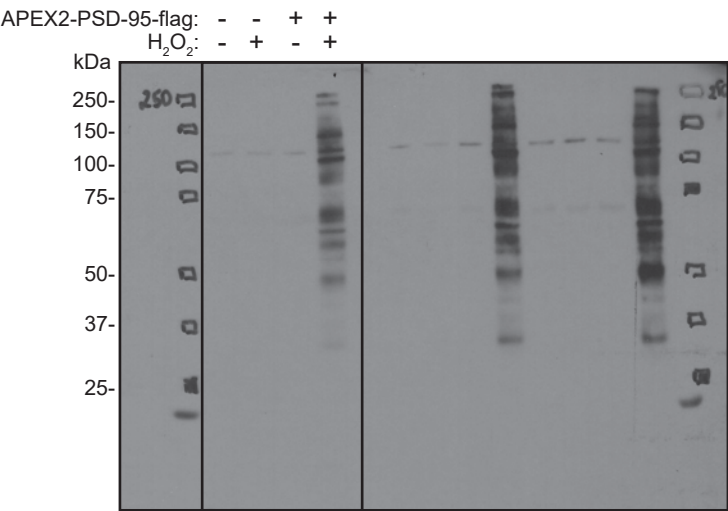

Blot #2: anti-flag

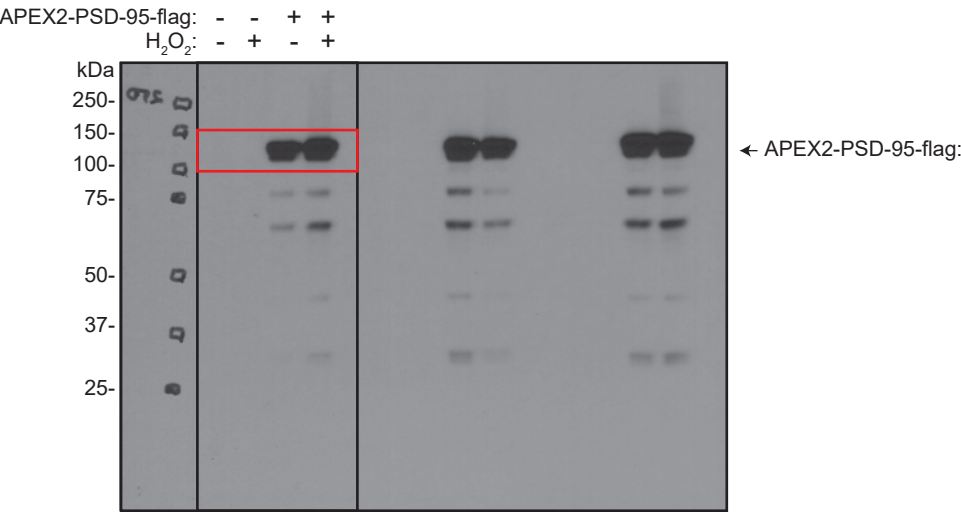

Blot #3: anti-GAPDH

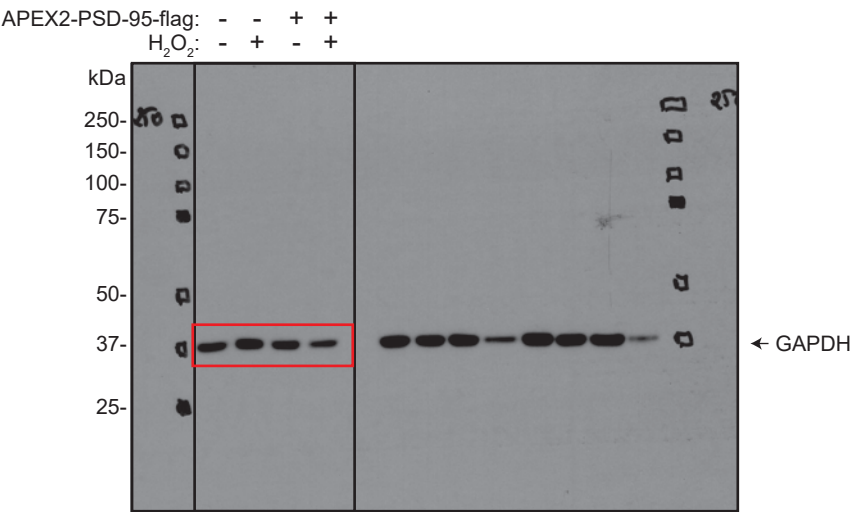

Supplement: Supplementary file 1 — Supplementary Material 1 [file 13024_2026_928_MOESM1_ESM.pdf]
