# Supplementary material for: Tau oligomers modulate synapse fate by eliciting progressive bipartite synapse dysregulation and synapse loss
Source: Mol Neurodegener. 2026 Jan 22;21:13. doi: 10.1186/s13024-026-00928-2 (PMC12918473; doi:10.1186/s13024-026-00928-2)
Supplement: Supplementary file 2 — Supplementary Material 2 [file 13024_2026_928_MOESM2_ESM.pdf]

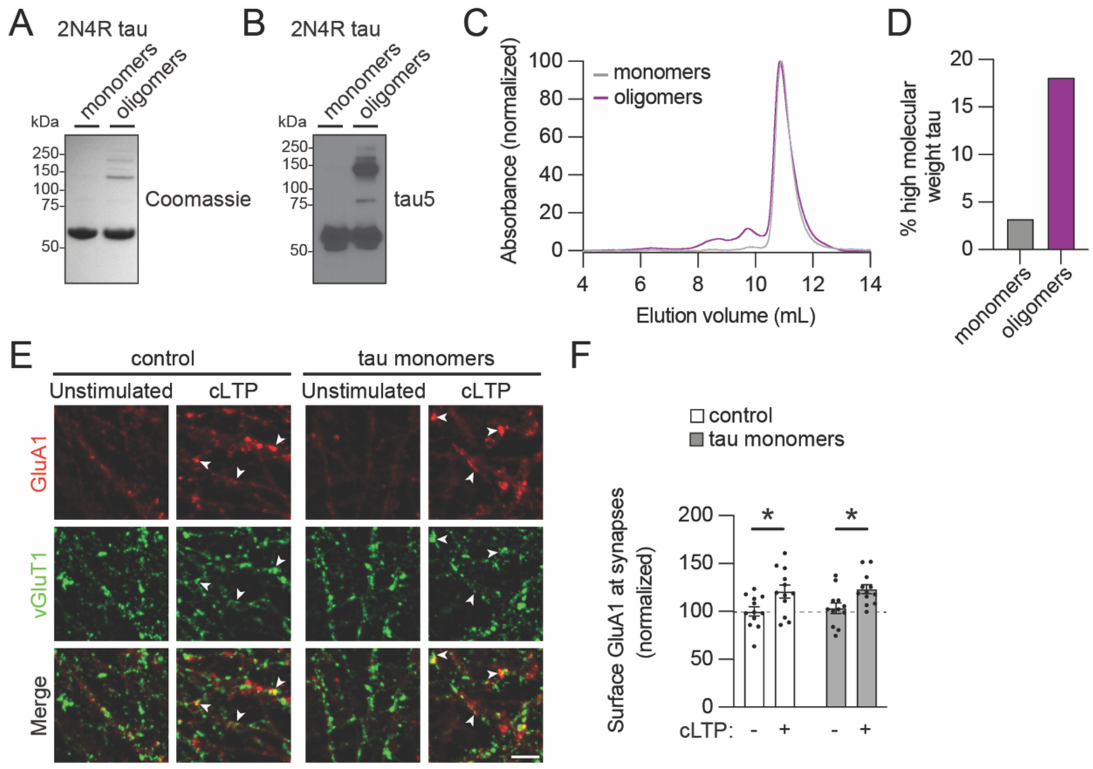

Supplemental Figure 1: Characterization of recombinant human tau oligomers.
(**A**) Non-reducing SDS-PAGE gel of preparations used for recombinant human 2N4R tau monomers and tau oligomers stained with Coomassie dye. The tau oligomer preparation contained tau oligomers, at the expected sizes of tau dimers and trimers, as well as tau monomers.
(**B**) Immunoblot with a tau5 antibody on preparations used for recombinant human 2N4R tau monomers and tau oligomers.
(**C**) Size exclusion chromatography (SEC) profiles of the preparations used for recombinant 2N4R tau monomers (gray) and tau oligomers (purple). Proteins eluted at earlier fractions (8 to 10 mL) correspond to high molecular weight tau, while tau monomers eluted at later fractions (10 to 12 mL). The absorbance was normalized to the highest peak.
(**D**) Quantification of the percentage of the tau preparations that were higher molecular weight.
(**E**) Representative confocal images of surface GluA1 (red) co-immunostaining with vGluT1 (green) to label surface AMPARs at synapses that are enhanced after cLTP induction (arrowheads) in human neurons exposed to tau monomers or a vehicle control for 30 min. Scale bar, 5 µm.
(**F**) Quantification of surface GluA1 immunolabeling intensity colocalized with vGluT1 at synapses in unstimulated and cLTP neurons (n = 12 neurons/group; * $p < 0.05$ two-way ANOVA, Bonferroni post hoc analyses).

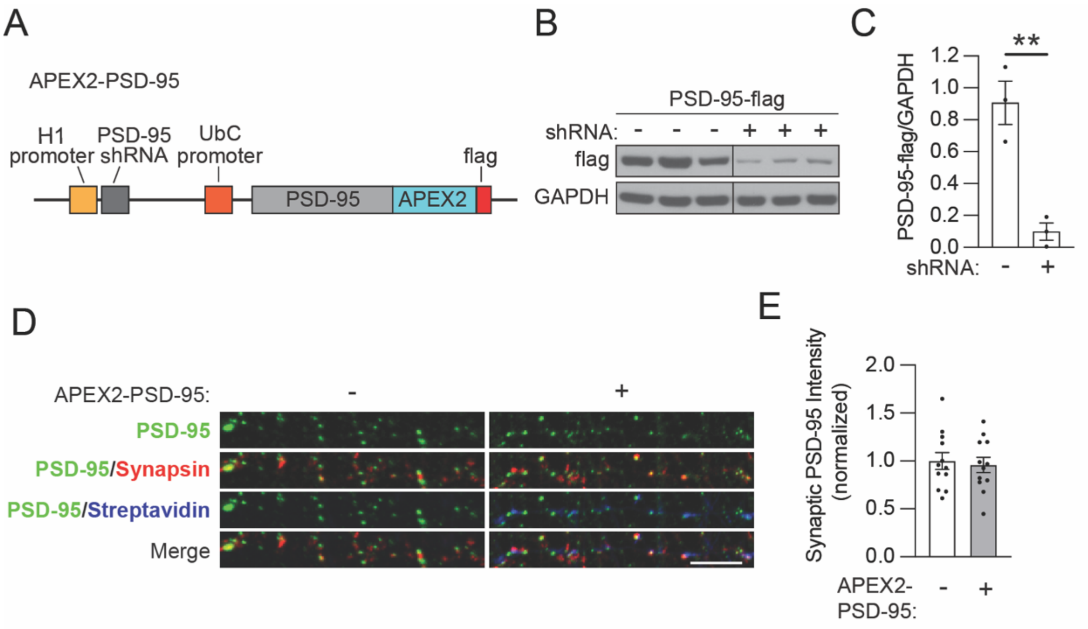

Supplemental Figure 2: APEX2-PSD-95 strategy of postsynaptic mapping.
(**A**) Design of the APEX2-PSD-95 construct containing a ubiquitin C promoter (UbC) to drive APEX2-PSD-95-flag expression and an H1 promoter to drive the expression of a short hairpin RNA (shRNA) to knockdown endogenous human PSD-95.
(**B**) Immunoblots of lysates from HEK293 cells expressing human flag-tagged PSD-95 with or without PSD-95 shRNA.
(**C**) Quantification of PSD-95-flag immunolabeling on the western blot from HEK293 cell lysates with or without PSD-95 shRNA expression normalized to GAPDH levels (n = 3 wells/treatment; ** $p < 0.01$, Unpaired Student's *t*-test).
(**D**) Representative images of PSD-95 (green) colocalized with Synapsin (red) in untransfected control neurons and neurons expressing APEX2-PSD-95. Streptavidin (blue) staining shows neurons that have APEX2-PSD-95-mediated biotinylation. This was done to confirm the analysis of total PSD-95 levels at synapses on neurons that had active APEX2-PSD95 (detected by Streptavidin) compared to uninfected neurons. Scale bar: 5 μm.
(**E**) Quantification of PSD-95 intensity levels colocalized with Synapsin in uninfected control neurons and neurons expressing APEX2-PSD-95. Values were normalized to the mean PSD-95 intensity in the uninfected control neurons (n = 12 images/group, not significant, Unpaired Student's *t*-test).

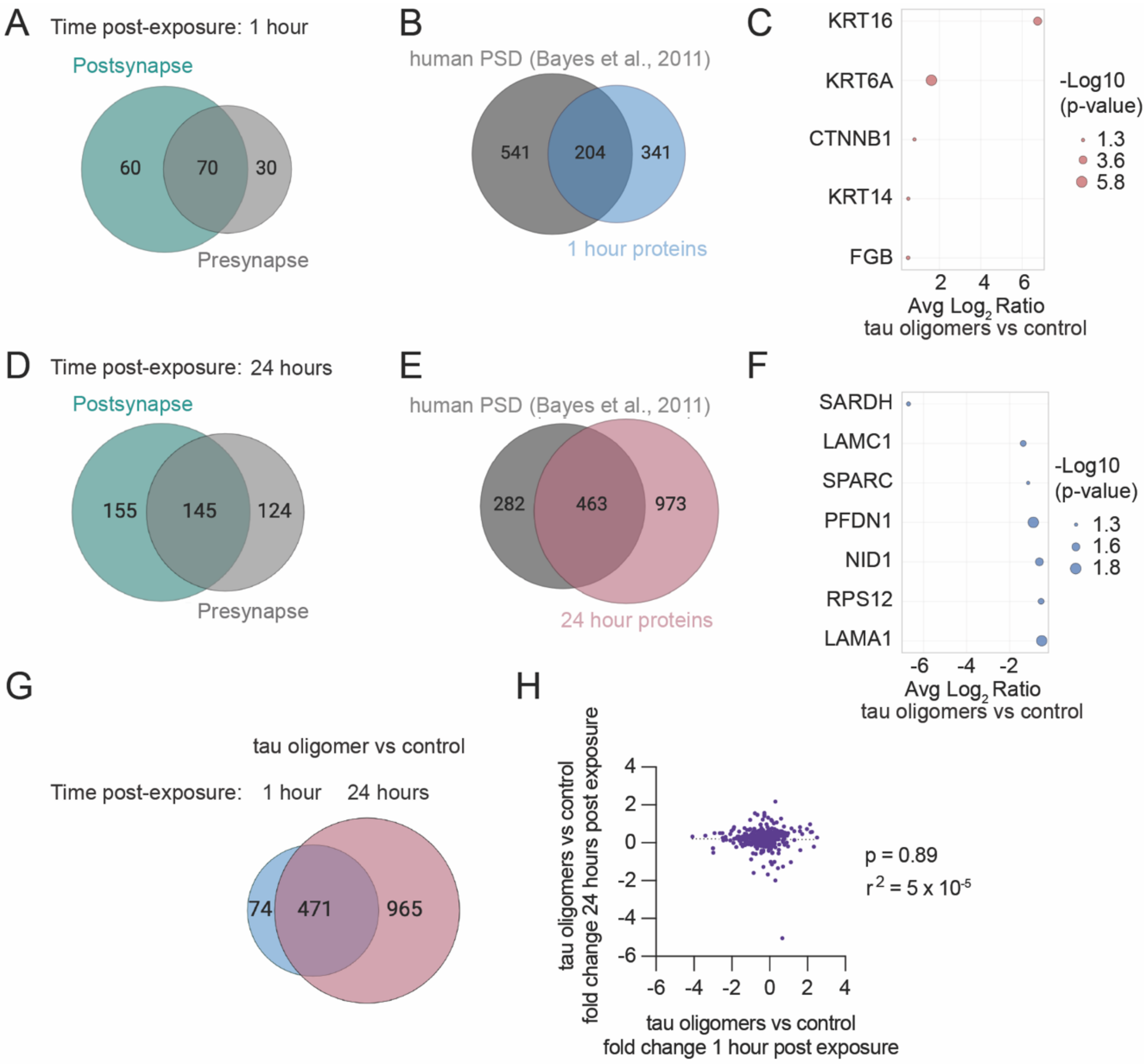

Supplemental Figure 3: Functional relationship and characterization of biotinylated proteins identified by mass spectrometry 1 hour and 24 hours after exposure of human neurons to tau oligomers.

(**A**) Venn diagram of biotinylated proteins categorized in postsynapse and presynapse SynGo cellular component analysis showed an overlap between 70 proteins in both presynaptic and postsynaptic compartments.

(**B**) Venn diagram revealed the overlap between the 545 biotinylated proteins detected in human neurons with APEX2-PSD-95-mediated proximity labeling and 745 proteins identified in the human postsynaptic density by mass spectrometry performed by Bayes et al., 2011.

(**C**) Graph of the 5 biotinylated proteins that were significantly upregulated 1 hour after in tau oligomer-exposed neurons.

(**D**) Venn diagram of annotated genes from SynGo cellular component analysis showing overlap between 300 postsynaptic genes and 269 presynaptic proteins.

(**E**) Venn diagram showing overlap between 1436 proteins identified by mass spectrometry 24 hours after exposure of human neurons to tau oligomers and 745 proteins from human postsynaptic density identified by mass spectrometry performed by Bayes et al., 2011.

(**F**) Graph of the 7 biotinylated proteins that were significantly downregulated 24 hours after in tau oligomer-exposed neurons.

(**G**) Venn diagram showing overlap between 545 and 1436 proteins identified by mass spectrometry 1 hour and 24 hours after exposure of human neurons to tau oligomers.

(**H**) Pearson correlation analyses of the relative changes in biotinylated proteins (Avg Log_2 Ratio) 1 hour and 24 hours after exposure of human neurons to tau oligomers. All quantifiable biotinylated proteins that were determined across both time points were plotted.

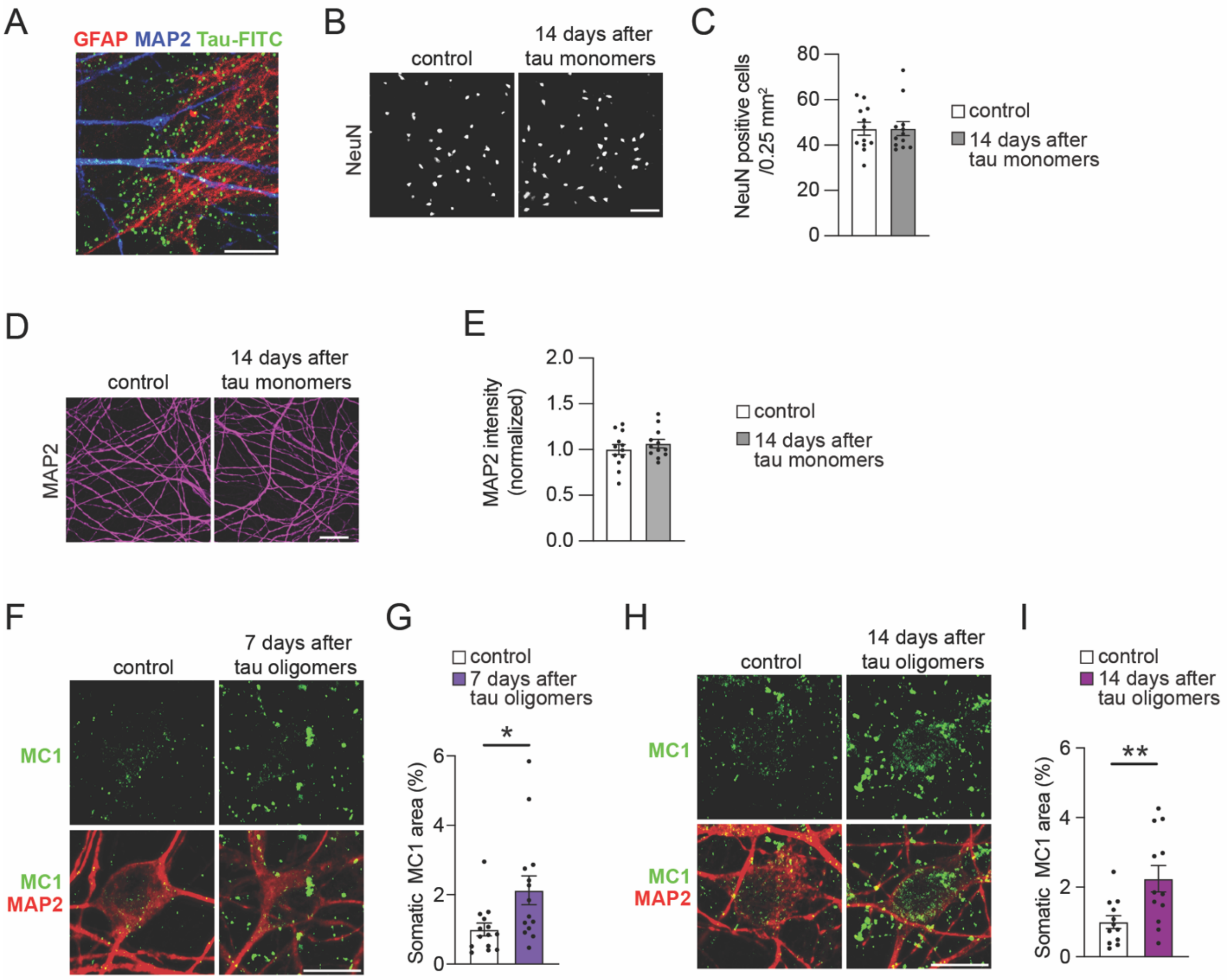

Supplemental Figure 4: Internalization of tau oligomers by astrocytes in the human neuron cultures and a prolonged increase in MC1-positive tau in the human neurons.

(**A**) Confocal image of human neurons treated with oligomerized tau-FITC for 30 min show colocalization of tau-FITC (green) with the astrocytic marker, GFAP (red). Scale bar: 10 µm.

(**B, C**) Representative images (**B**) and quantification (**C**) of NeuN immunolabeling in vehicle-treated control neurons and in neurons 14 days after the 30-min exposure to tau monomers (n = 12 images/group; no significant difference, Unpaired Student's *t*-test). Scale bar: 100 mm.

(**D, E**) Representative confocal images of MAP2 immunostaining in neurons at 14 days after the 30-min exposure to tau monomers and in neurons treated with vehicle control. Scale bars: 20 µm. The intensity of MAP2 immunolabeling in dendrites of human neurons was quantified (n = 12 images/group; no significant difference, Unpaired Student's *t*-test)

(**F-I**) Representative confocal images of MAP2 (red) and MC1 (green) immunostaining in human neurons at (**F**) 7 or (**H**) 14 days after tau oligomer exposure. Scale bars: 10 µm. Quantification of the percent area occupied by MC1 immunolabeling in the soma of human neurons at (**G**) 7 days (n = 14 images/group; * $p < 0.05$, Unpaired Student's *t*-test) and (**I**) 14 days (n = 12 images/group; ** $p < 0.01$ Unpaired Student's *t*-test) after tau oligomer exposure. Values are given as means ± SEM.

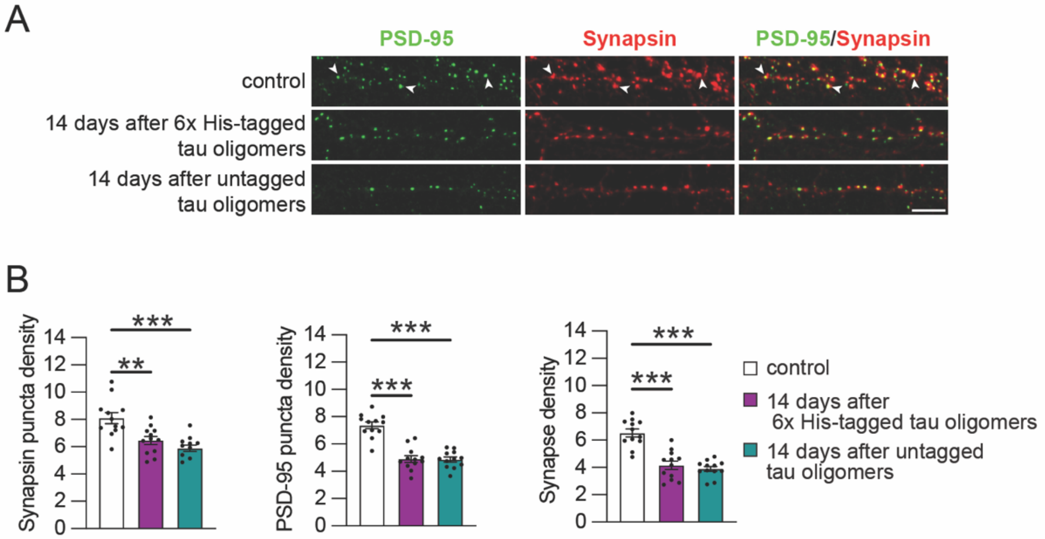

Supplemental Figure 5: Acute exposure to untagged or 6x His-tagged tau oligomers causes similar synapse loss.

(**A**) Representative confocal images of presynaptic Synapsin (red) colocalized with postsynaptic PSD-95 (green) in human neurons 14 days after exposure to vehicle, 6x His-tagged tau oligomers or untagged tau oligomers for 30 min (n = 12 images/group; Unpaired Student's *t*-test). Scale bar: 5 µm.

(**B**) Quantification of the density of individual Synapsin, PSD-95 puncta and colocalized Synapsin and PSD-95 to assess synapse density along neuronal processes (# of puncta/10 µm length) (n = 12 images/group; ** $p < 0.01$, *** $p<0.001$, one-way ANOVA, Bonferroni post hoc analyses).

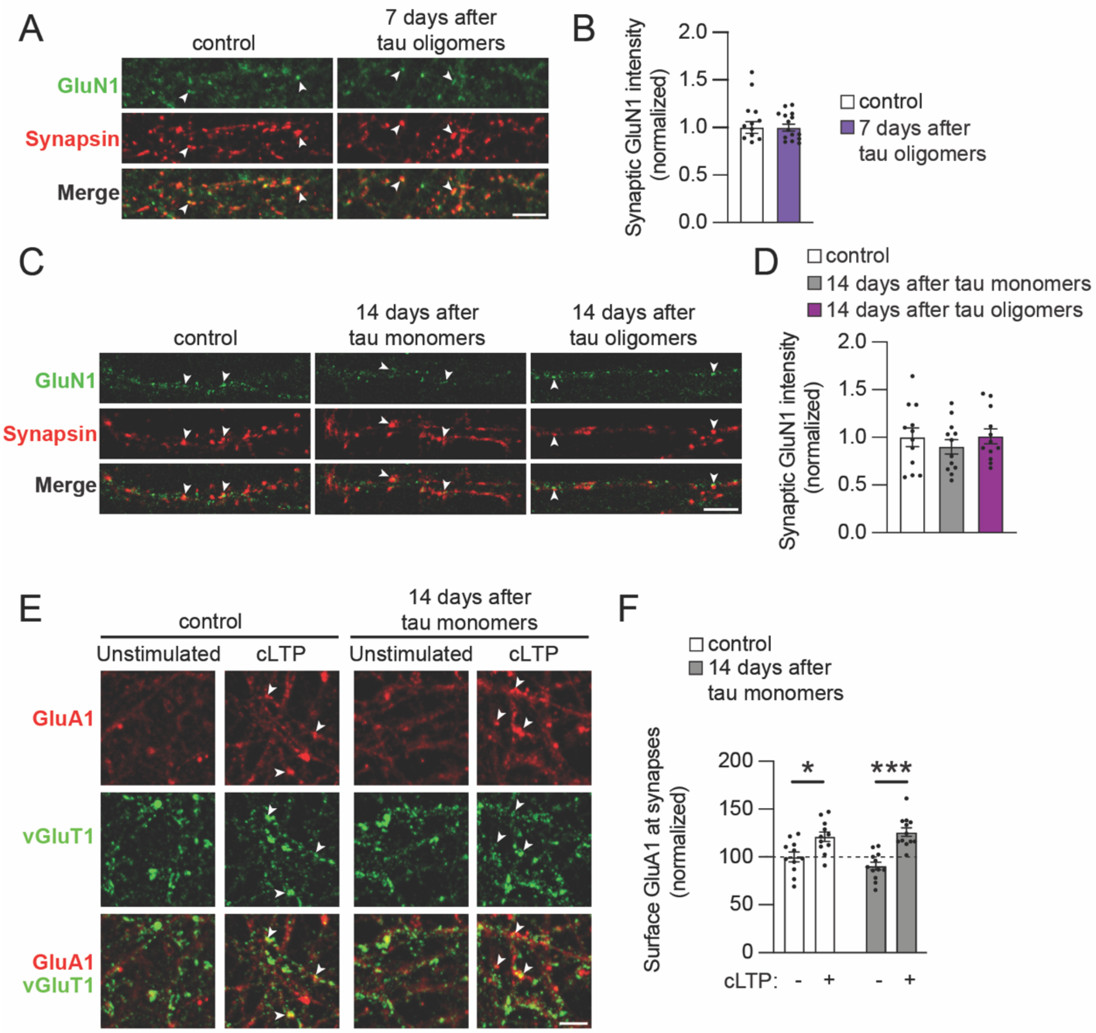

Supplemental Figure 6: Acute tau oligomer exposure does not alter NMDAR levels at synapses in human neurons.

(**A, B**) Representative confocal images (**A**) and quantification (**B**) of GluN1 (green) colocalized with the presynaptic marker Synapsin (red, arrowheads) in human neurons 7 days after exposure to tau oligomers for 30 min (n = 12-15 images/group; no significant difference, Unpaired Student's *t*-test). Scale bar: 5 µm.

(**C, D**) Representative confocal images (**C**) and quantification (**D**) of GluN1 (green) colocalized with the presynaptic marker Synapsin (red) in human neurons 14 days after exposure to vehicle, tau monomers or tau oligomers for 30 min (n = 12 images/group; no significant difference, one-way ANOVA, Bonferroni post hoc analyses). Scale bar: 5 µm.

(**E, F**) Representative confocal images (**E**) and quantification (**F**) of surface GluA1 immunolabeling (red) colocalized with vGluT1 immunolabeling (green) of synapses on neurons that were unstimulated or after cLTP induction (arrowheads). Neurons were either exposed to tau monomers for 30 min or vehicle control, and they were fixed for immunolabeling 14 days later. All values are normalized to unstimulated control neurons (n = 12 images/group, two-way ANOVA, Bonferroni post hoc analyses). Scale bar: 5 µm.

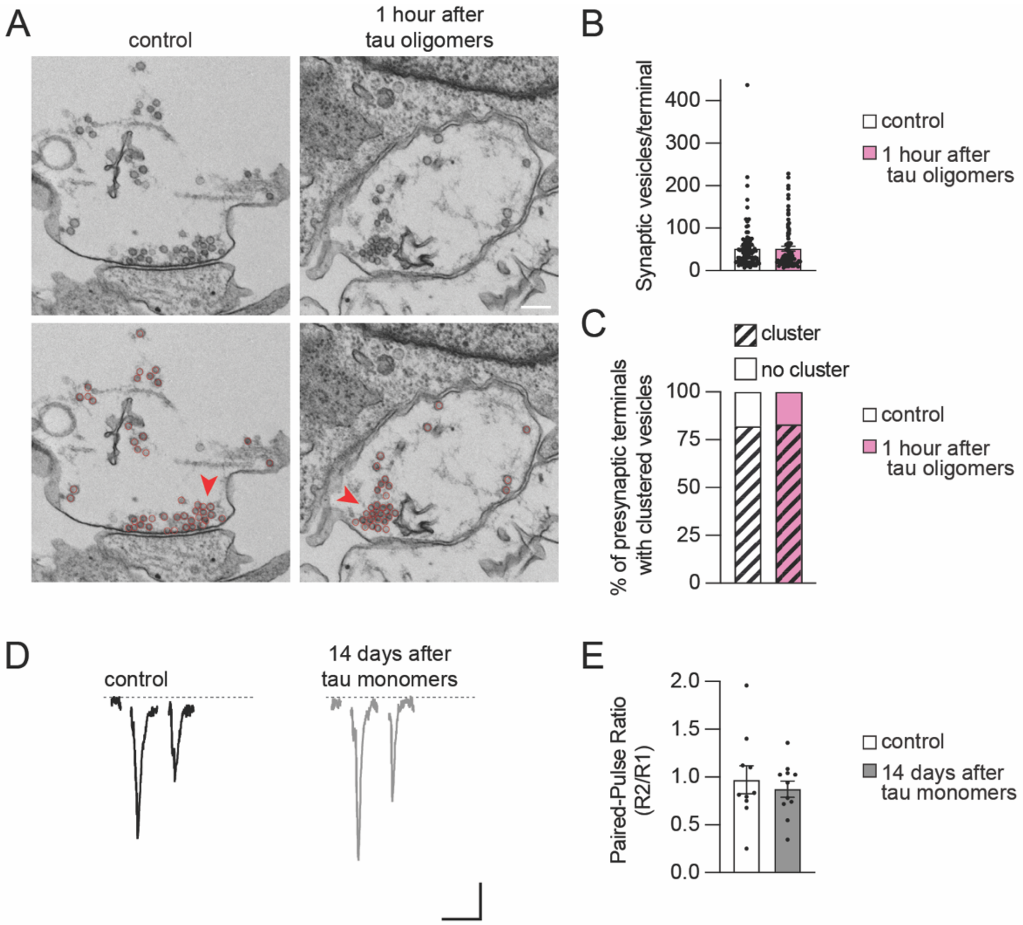

Supplemental Figure 7: Short-term effect of acute tau oligomer exposure on presynaptic vesicle organization and tau monomer effect on vesicle release probability.

(**A**) Representative electron micrograph of human neuron synapses 1 hour after treatment with either tau oligomers or vehicle for 30 min (top). The respective identical images below depict the synaptic vesicles highlighted by red circles and red arrows point to clustered vesicles. Scale bar: 200 nm. Magnification: 25,000 x.

(**B**) Graph of the number of synaptic vesicles quantified in each presynaptic terminal of the human neurons with and without tau oligomer exposure (n = 88-94 synapses/group; no significant difference, Unpaired Student's *t*-test).

(**C**) Graph of the percentage of presynaptic terminals at human neuron synapses that contained vesicle clusters (number of terminals with clustered vesicles/total synapses; control:77/94; 1 hour:83/88).

(**D**) Representative traces from patch-clamp recordings in human neurons elicited by two consecutive extracellular field stimuli (15 µA) with a 20 ms interstimulus interval (ISI). Recordings were performed 14 days after human neurons were exposed to vehicle control (black) or tau monomers (gray) for 30 min. Scale bars: 25 pA, 20 ms.

(**E**) The paired-pulse ratio was calculated at the 20 ms ISI by dividing the amplitude of the second evoked EPSC (R2) by the amplitude of the first evoked EPSC (R1) (n = 10-11 cells/group; no significant difference, Unpaired Student's *t*-test).
