# Supplementary material for: Tau oligomers modulate synapse fate by eliciting progressive bipartite synapse dysregulation and synapse loss
Source: Mol Neurodegener. 2026 Jan 22;21:13. doi: 10.1186/s13024-026-00928-2 (PMC12918473; doi:10.1186/s13024-026-00928-2)

Supplemental Figure 1A

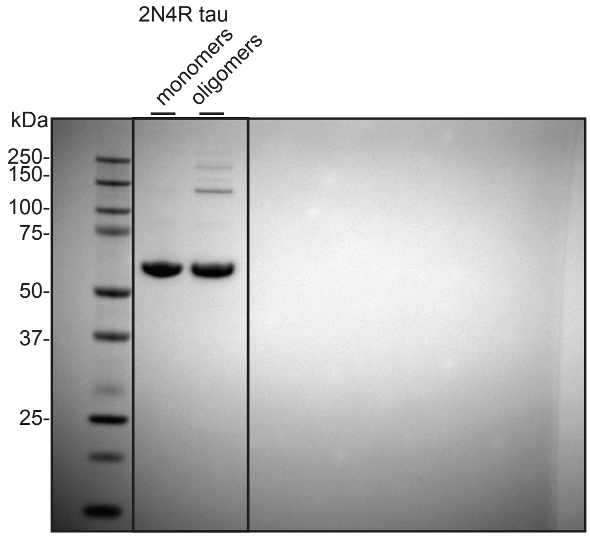

Supplemental Figure 1B

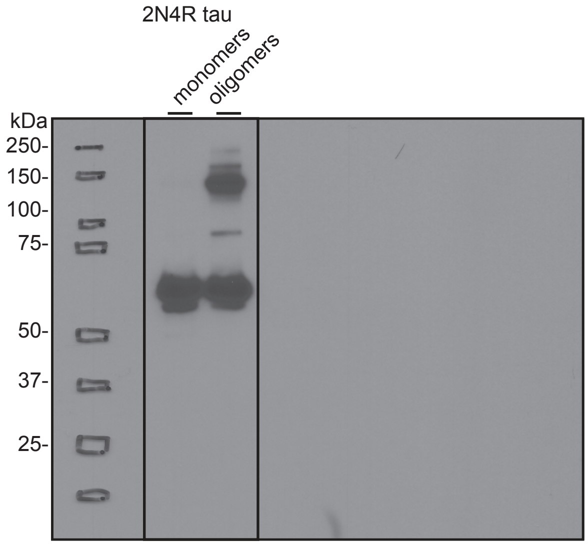

Supplemental Figure 2B

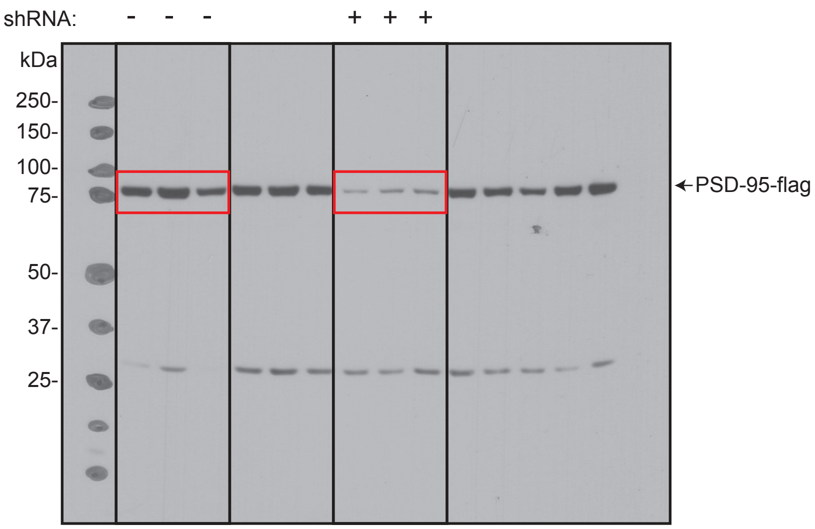

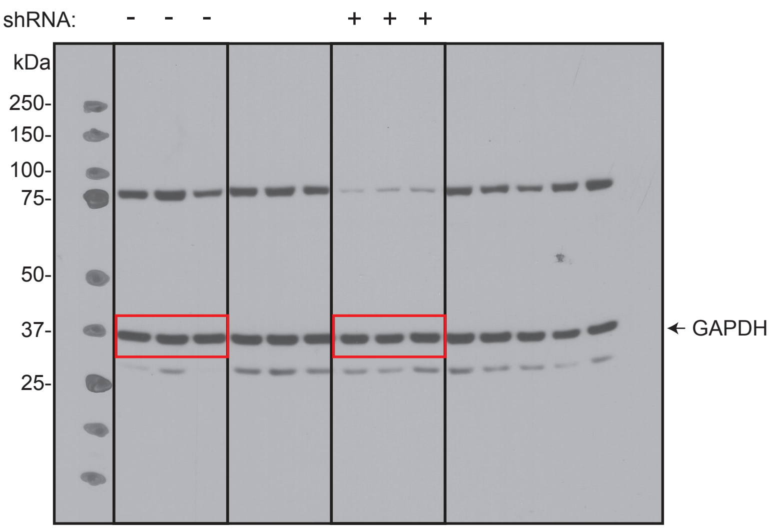

Supplement: Supplementary file 3 — Supplementary Material 3 [file 13024_2026_928_MOESM3_ESM.pdf]
